# Effects of poly (ADP-ribose) polymerase inhibitor treatment on the repair process of ischemic acute kidney injury

**DOI:** 10.1038/s41598-023-50630-2

**Published:** 2024-01-02

**Authors:** Junseok Jeon, Kyungho Lee, Hye Ryoun Jang, Kyeong Eun Yang, Cheol-Jung Lee, Hyeonju Ahn, Woong-Yang Park, Jung Eun Lee, Ghee Young Kwon, Yoon-Goo Kim, Wooseong Huh

**Affiliations:** 1 Division of Nephrology, Department of Medicine, Samsung Medical Center, Sungkyunkwan University School of Medicine, Seoul, Republic of Korea; 2 Division of Scientific Instrumentation and Management, Korea Basic Science Institute, Daejeon, Republic of Korea; 3 Samsung Genome Institute, Samsung Medical Center, Seoul, Republic of Korea; 4 Innovative Institute for Precision Medicine, Samsung Medical Center, Seoul, Republic of Korea; 5 Department of Pathology, Samsung Medical Center, Sungkyunkwan University School of Medicine, Seoul, Republic of Korea

**Keywords:** Acute kidney injury, Inflammation

## Abstract

Excessive activation of poly (ADP-ribose) polymerase (PARP) contributes to ischemic acute kidney injury (AKI). PARP inhibition has been shown to be beneficial in renal ischemia–reperfusion injury (IRI) in the early phase, but its role in the repair process remains unclear. The effects of JPI-289, a novel PARP inhibitor, during the healing phase after renal IRI were investigated. IRI was performed on 9-week-old male C57BL/6 mice. Saline or JPI-289 100 mg/kg was intraperitoneally administered once at 24 h or additionally at 48 h after IRI. Hypoxic HK-2 cells were treated with JPI-289. Renal function and fibrosis extent were comparable between groups. JPI-289 treatment caused more prominent tubular atrophy and proinflammatory intrarenal leukocyte phenotypes and cytokines/chemokines changes at 12 weeks after unilateral IRI. JPI-289 treatment enhanced gene expressions associated with collagen formation, toll-like receptors, and the immune system in proximal tubules and endothelial cells after IRI. JPI-289 treatment at 3 or 6 h after hypoxia facilitated proliferation of hypoxic HK-2 cells, whereas further treatment after 24 h suppressed proliferation. Delayed inhibition of PARP after renal IRI did not facilitate the repair process during the early healing phase but rather may aggravate renal tubular atrophy during the late healing phase in ischemic AKI.

## Introduction

Ischemic acute kidney injury (AKI) is the most common cause of AKI and can be precipitated by several clinical conditions that reduce renal blood flow, including kidney transplantation^1^. AKI is of great clinical importance because it not only increases overall mortality but also promotes the development and progression of chronic kidney disease^2^. Ischemia–reperfusion injury (IRI) is defined as the paradoxical exacerbation of tissue damage following the restoration of blood flow to ischemic tissues; it triggers a series of deleterious cellular responses in the affected organ^3^. A robust inflammatory response follows IRI, and this immunologic mechanism is one of the main pathogeneses of ischemia AKI^4,5^. Numerous interventions aimed at relieving renal IRI have been evaluated in animal studies but none have been proven to be effective enough for clinical application^3^.

Poly (ADP-ribose) polymerase 1 (PARP-1) is known to play important roles in DNA repair and the maintenance of genome integrity. However, excessive activation of PARP by severe DNA damage consumes NAD +, resulting in energy depletion, cell death, and necrosis^6^. PARP-1 also regulates the expression of various proteins, such as NF-κB, associated with inflammation at the transcriptional level^7,8^. Activation of PARP has been demonstrated in the ischemic injury of various tissues, including kidney^9–11^. PARP ablation or inhibition preserved ATP levels and renal functions and attenuated the inflammatory response after IRI in the mouse model^11,12^. JPI-289, a recently developed novel PARP-1 inhibitor with potent PARP-1 inhibitory activity, revealed beneficial effects in ischemic stroke models^13,14^. We recently reported that PARP inhibition by JPI-289 treatment immediately after renal IRI attenuated renal injury and intrarenal inflammation in the murine ischemic AKI model^15^.

Although PARP-1 is involved in DNA repair and various transcription processes, it remains uncertain whether PARP inhibitors will have beneficial effects beyond the early injury phase. Most studies, including the ischemic AKI model, either treated animals with PARP inhibitors before or immediately after tissue injury or used a genetically PARP ablation model; therefore, the effects of PARP inhibitors on the healing phase after tissue injury remain unknown^11–15^. This study investigated the effects of pharmacologic PARP-1 inhibition with late JPI-289 treatment after the establishment of renal IRI on the postischemic kidney.

## Results

### Effects of JPI-289 treatment on renal function after renal IRI

**Figure 1:**
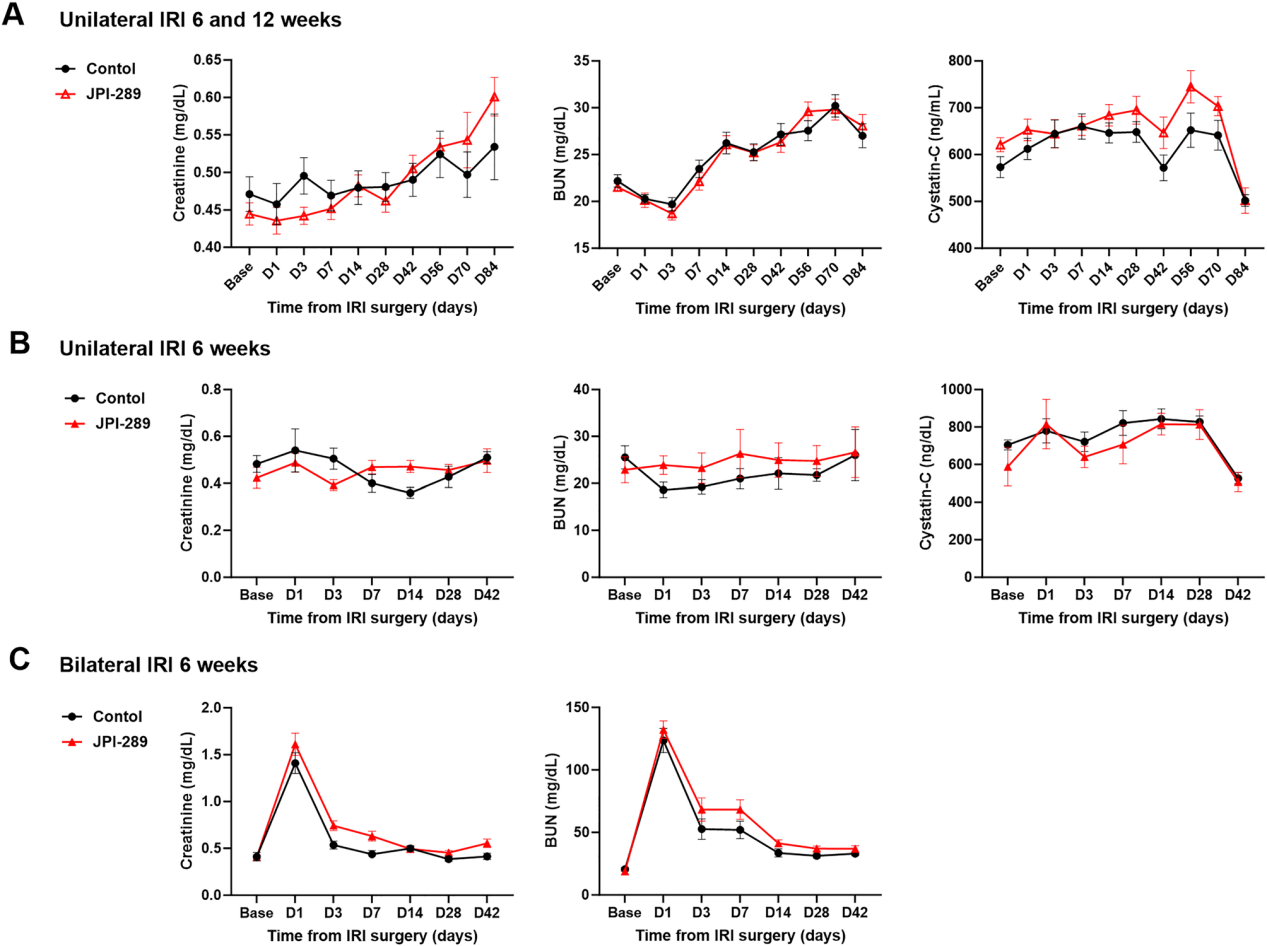
Renal function following IRI. (**A**) Treatment with JPI-289 100 mg/kg at 24 and 48 h twice versus control after unilateral IRI. (**B**) Treatment with JPI-289 100 mg/kg at 24 h once versus control after unilateral IRI. (**C**) Treatment with JPI-289 100 mg/kg at 24 h once versus control after bilateral IRI.

After unilateral IRI, BUN, plasma creatinine, and cystatin C were comparable between the JPI-289 treated and control groups (Fig. 1A,B). Despite bilateral IRI, there were no differences in BUN and plasma creatinine between the JPI-289 treated and control groups (Fig. 1C).

### Effects of JPI-289 treatment on histological changes after renal IRI

**Figure 2:**
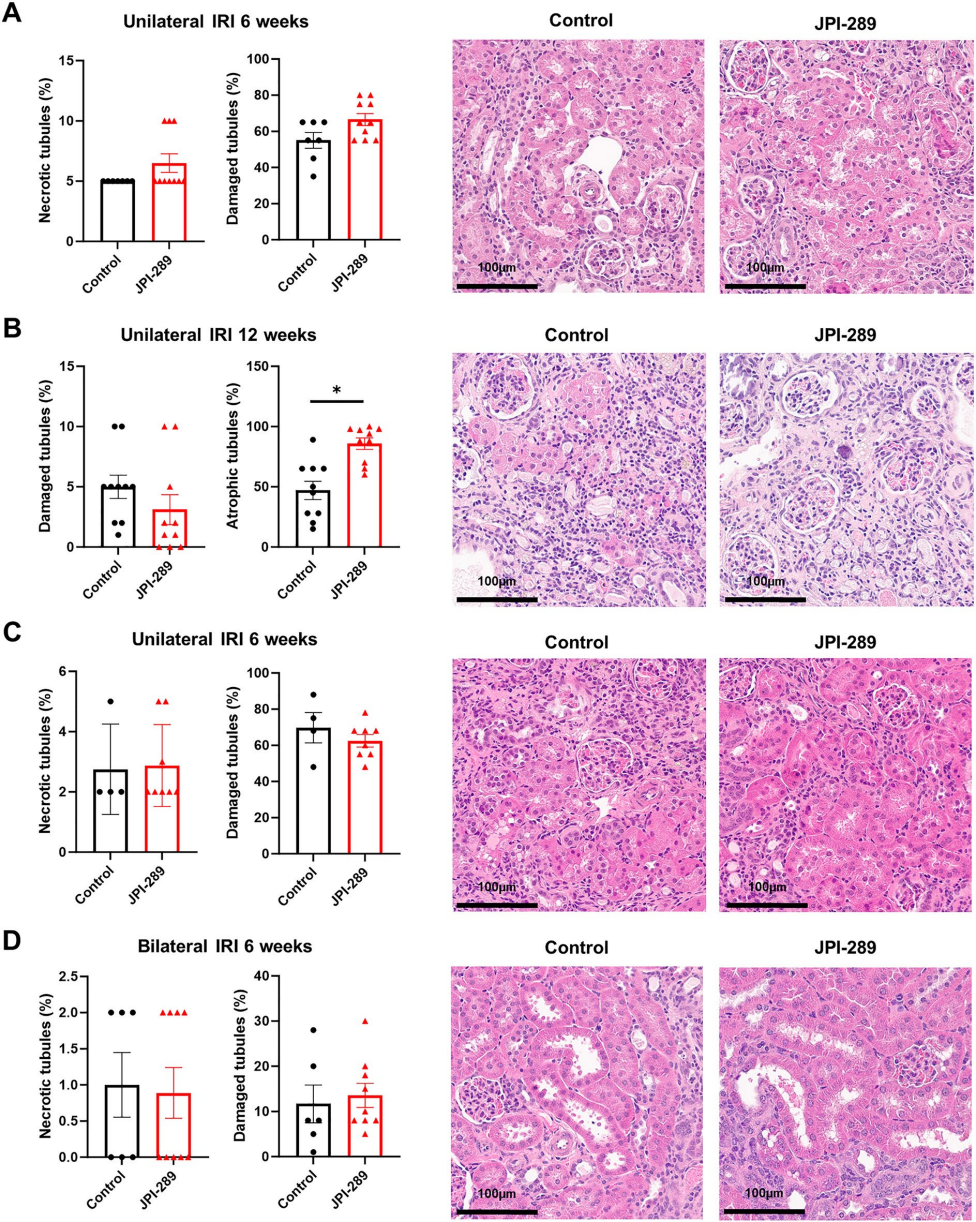
Renal tubular necrosis, damage, or atrophy after renal IRI. (**A**) Treatment with JPI-289 100 mg/kg at 24 and 48 h twice versus control after unilateral IRI (6 weeks). (**B**) Treatment with JPI-289 100 mg/kg at 24 and 48 h twice versus control after unilateral IRI (12 weeks). (**C**) Treatment with JPI-289 100 mg/kg at 24 h once versus control after unilateral IRI. (**D**) Treatment with JPI-289 or 100 mg/kg at 24 h once versus control after bilateral IRI.

The proportion of atrophic tubules was higher in the JPI-289 100 mg/kg twice-treated group than in the control group at 12 weeks after unilateral IRI (Fig. 2B). Renal tubular necrosis or damage was comparable between the two groups at 6 weeks after bilateral or unilateral IRI (Fig. 2A,C, and D). The area of renal fibrosis was not different between the groups (Supplementary Fig. S1).

### Effects of JPI-289 treatment on leukocytes trafficking into postischemic kidney

**Figure 3:**
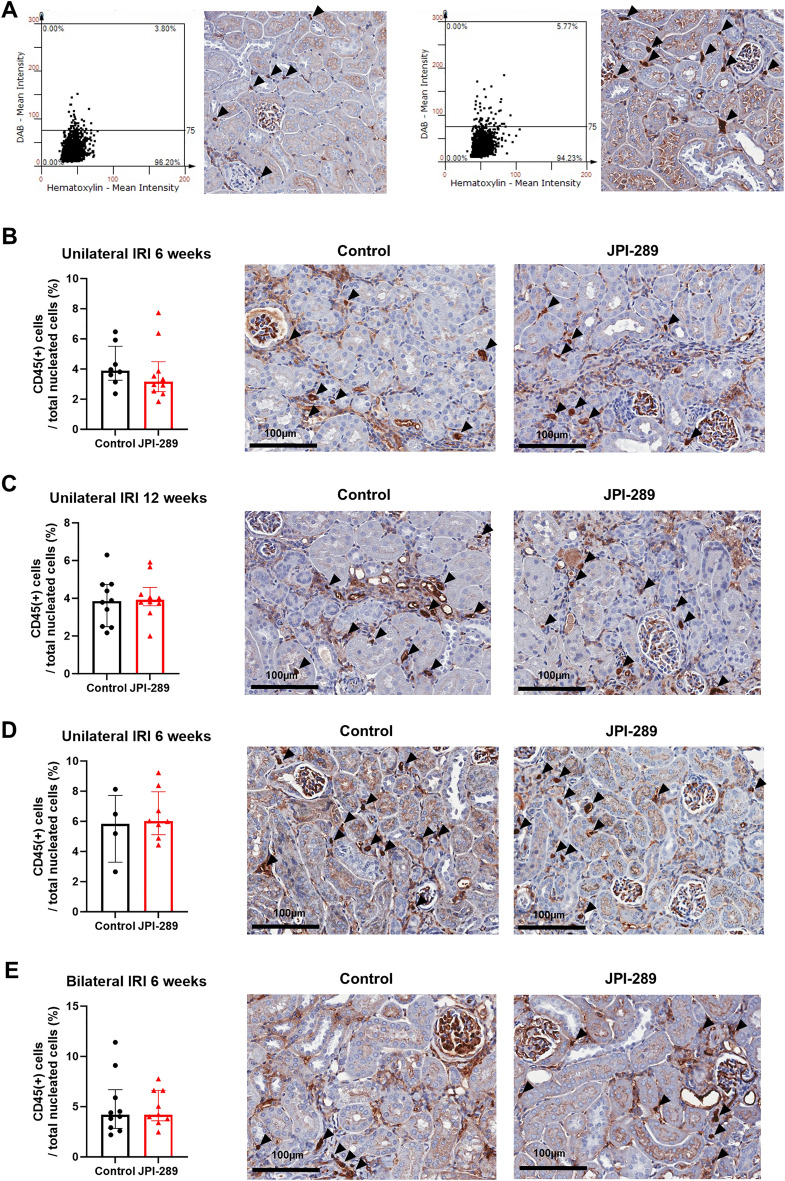
Leukocyte trafficking into the postischemic kidneys. (**A**) The examples of semiquantitative analysis of CD45-positive leukocytes in the postischemic kidney with relatively lower and higher percentage of CD45-positive leukocytes using automated imaging analysis system (TissueFAXS). Arrowheads indicate CD45-positive leukocytes (× 250). The whole fields of slides including both cortex and medulla were evaluated. (**B** and **C**) Treatment with JPI-289 100 mg/kg at 24 and 48 h twice versus control after unilateral IRI. (**D**) Treatment with JPI-289 100 mg/kg at 24 h once versus control after unilateral IRI. (**E**) Treatment with 100 mg/kg at 24 h once versus control after bilateral IRI.

The trafficking of total leukocytes into the postischemic kidney was evaluated using immunohistochemical staining of CD45. The proportion of total leukocytes expressing CD45 among total nuclei in the whole field of each slide was semiquantitatively calculated with the TissueFAXS system (Fig. 3A). Intrarenal total leukocytes expressing CD45 did not differ between the groups after bilateral or unilateral IRI (Fig. 3B,C,D and E).

### Effects of JPI-289 treatment on subtypes of intrarenal leukocytes after renal IRI

**Figure 4:**
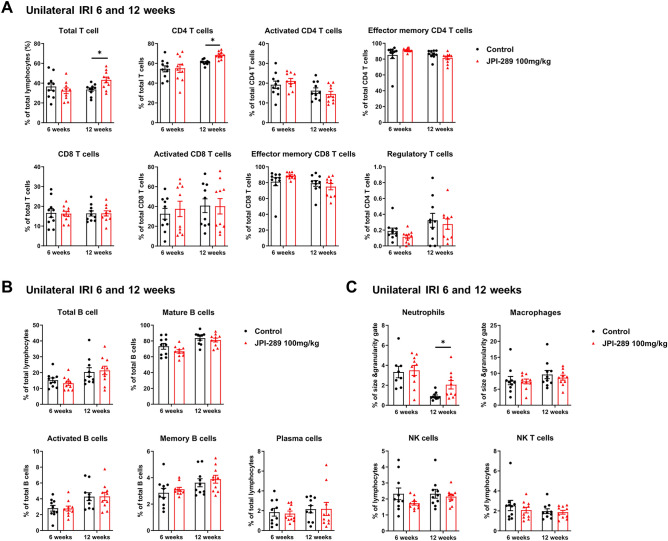
Flow cytometry analyses of intrarenal leukocyte subtypes between treatment with JPI-289 100 mg/kg at 24 and 48 h versus control after unilateral IRI. (**A**) T cell subtypes, (**B**) B cell subtypes, (**C**) Neutrophils, Macrophages, NK cells, NK T cells.

The phenotypes of kidney monounclear cells (KMNCs) in the postischemic kidneys were evaluated using flow cytometry. Supplementary Fig. S2 shows gating strategies. In the experiments of JPI 289 100 mg/kg twice treatment after unilateral IRI, the leukocyte subtypes were not different between the groups at 6 weeks after IRI (Fig. 4). The percentages of total T cells, CD4 T cells, and neutrophils were higher in the JPI-289 100 mg/kg twice-treated group compared to the control group at 12 weeks after unilateral IRI. The percentages of CD8 T cells, B cells, NK T cells, NK cells, and macrophages were not different between the groups at 12 weeks after unilateral IRI.

The percentages of total B cells in the JPI-289 100 mg/kg once-treated group after bilateral IRI and memory B cells in the JPI-289 100 mg/kg once-treated group after unilateral IRI were lower compared to the control groups of each model (Supplementary Fig. S3 and S4). The percentage of other subtypes of leukocytes was not different between the JPI-289 100 mg/kg once-treated and control groups at 6 weeks after bilateral or unilateral IRI.

### Effects of JPI-289 treatment on cytokines and chemokines expression after renal IRI

**Figure 5:**
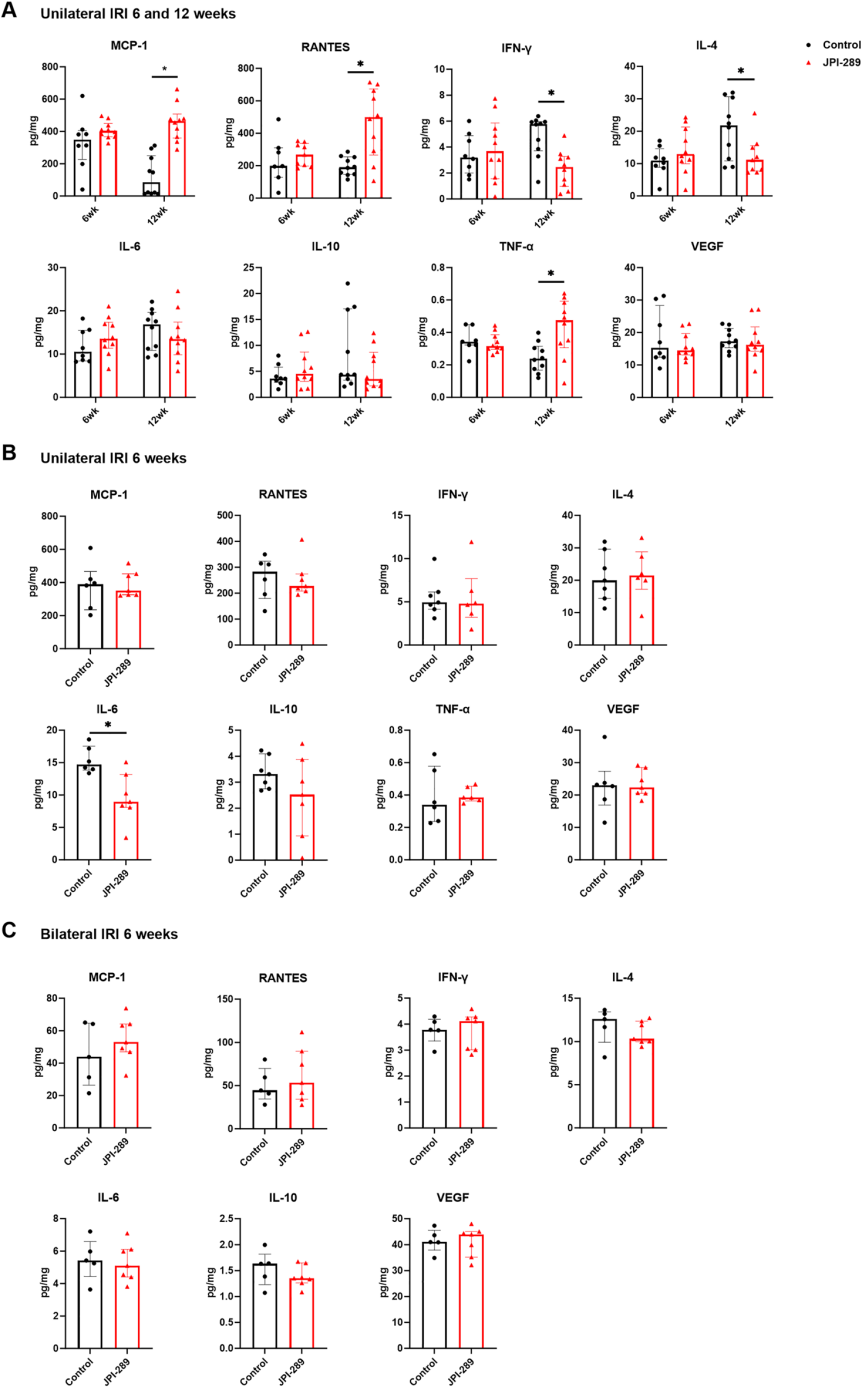
The expression of intrarenal cytokines and chemokines in the postischemic kidneys. (**A**) Treatment with JPI-289 100 mg/kg at 24 and 48 h twice versus control after unilateral IRI. (**B**) Treatment with JPI-289 100 mg/kg at 24 h once versus control after unilateral IRI. (**C**) Treatment with JPI-289 100 mg/kg at 24 h once versus control after bilateral IRI.

Intrarenal expression of MCP-1, RANTES, and TNF-α were enhanced, while the expression of IFN-*γ* and IL-4 were suppressed in the JPI-289 100 mg/kg twice-treated group compared to the control group at 12 weeks after unilateral IRI (Fig. 5A). The expression of IL-6 was suppressed in the JPI-289 100 mg/kg once-treated group compared to the control group at 6 weeks after unilateral IRI (Fig. 5B). There was no difference in intrarenal cytokines/chemokines expression at 6 weeks after bilateral IRI (Fig. 5C).

### The effects of JPI-289 on single-cell RNA expression of postischemic proximal tubules and endothelial cells

**Figure 6:**
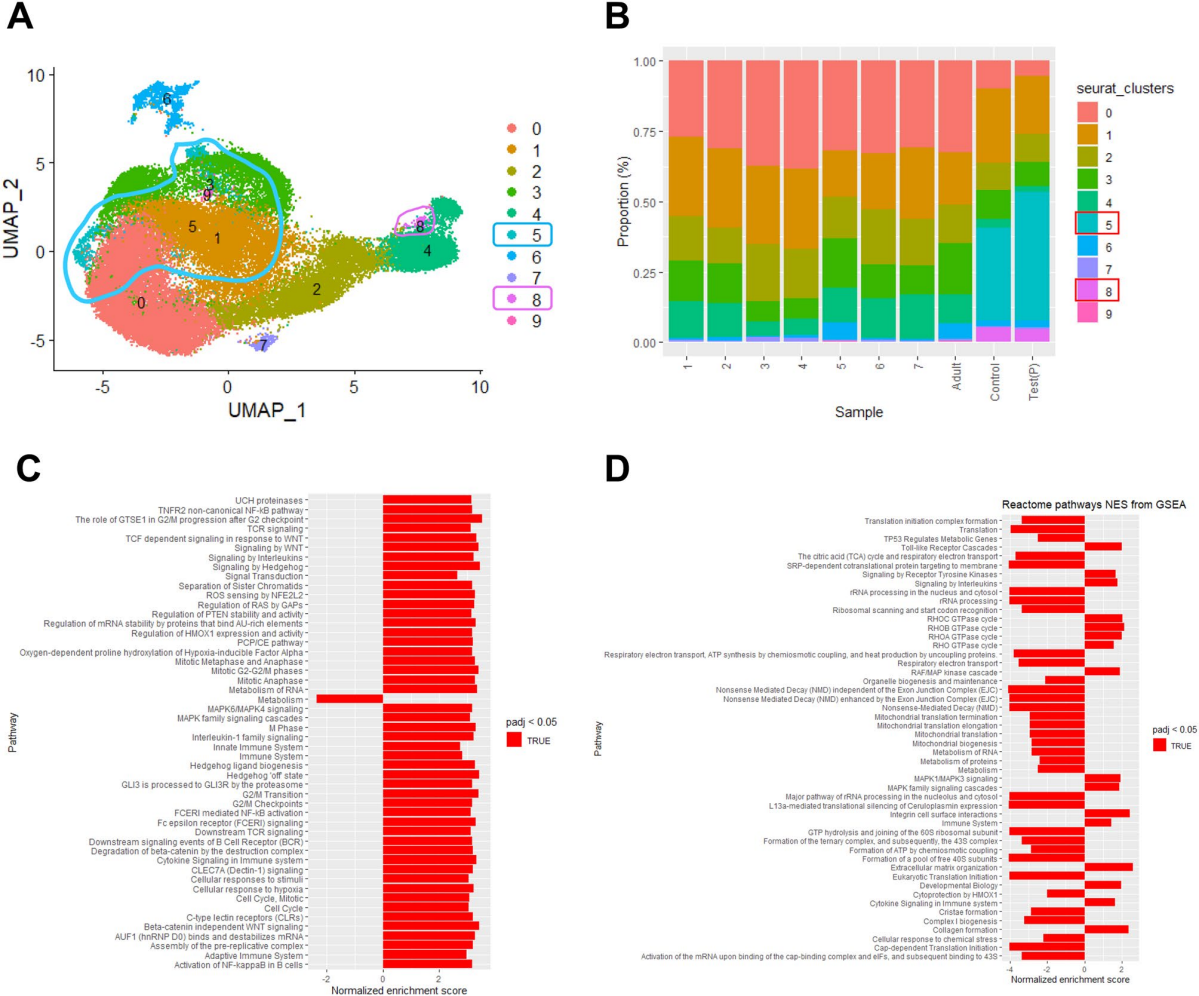
scRNA-seq results for proximal tubules of postischemic mouse kidney tissues (**A**) Uniform Manifold Approximation** (**UMAP) embedding of scRNA-seq profiles of proximal tubules, colored according to cells. (**B**) Proportions of cells of different subsets for each sample. Samples 1–7 and adult samples are mouse normal kidney samples of GSE107585 and GSE157079 acquired from the National Center for Biotechnology Information Gene Expression Omnibus (NCBI GEO) database. Test (P) and control sample represent sample of JPI-289 treated and control post-ischemic mouse kidney, respectively. Boxed clusters (clusters 5 and 8) indicate increased clusters in post-ischemic kidney compared to normal kidney. (**C**) Reactome pathways normalized enrichment score (NES) from gene set enrichment analysis (GSEA) of cluster 5. A positive NES indicates that genes in the JPI-289 treated sample are more expressed than those in the control sample and a negative indicates the opposite. (**D**) Reactome pathways NES from GSEA of cluster 8.

**Figure 7:**
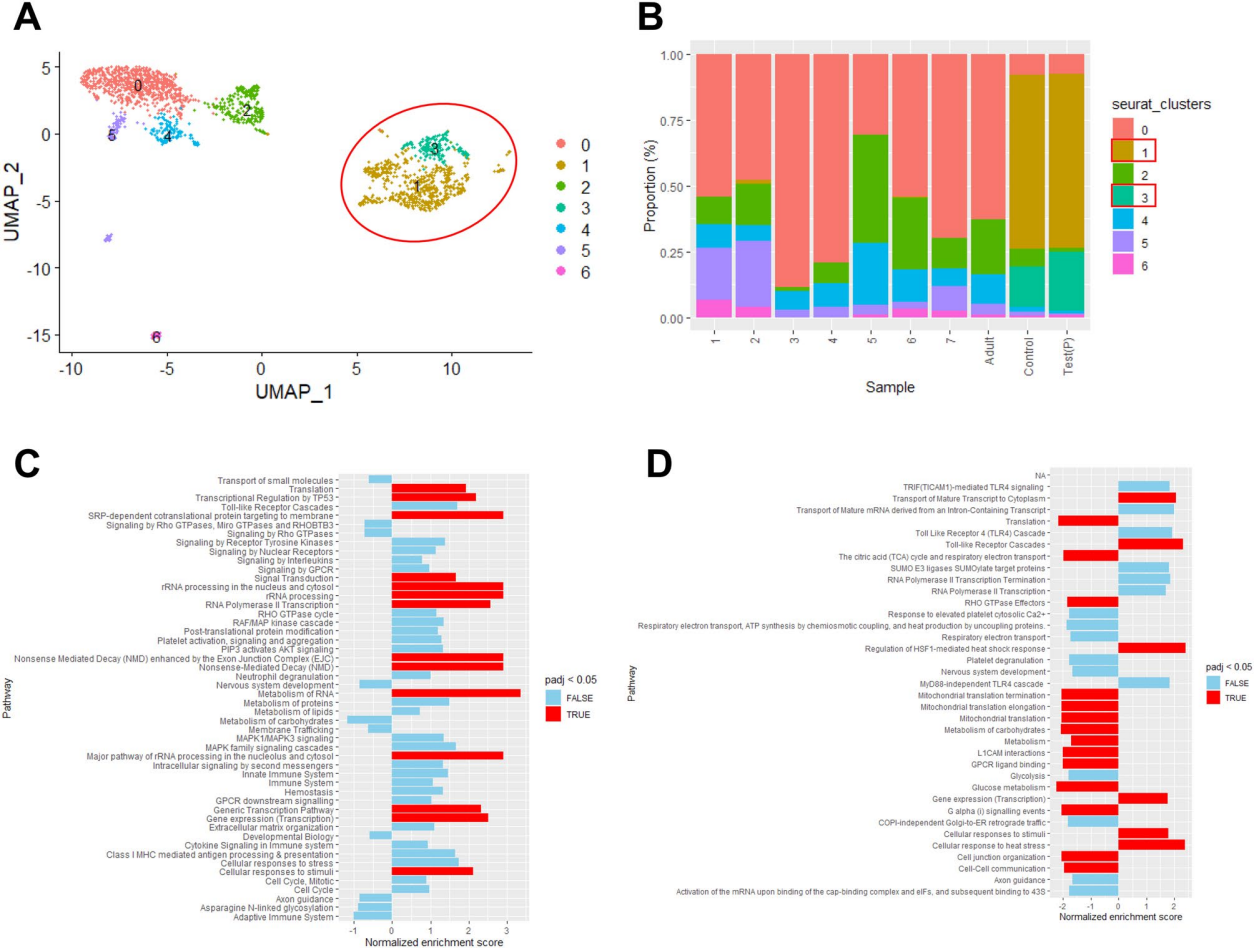
scRNA-seq results for endothelial cells of postischemic mouse kidney tissues (**A**) Uniform Manifold Approximation** (**UMAP) embedding of scRNA-seq profiles of endothelial cells, colored according to cells. (**B**) Proportions of cells of different subsets for each sample. Samples 1–7 and adult sample are normal mouse kidney samples of GSE107585 and GSE157079 acquired from the National Center for Biotechnology Information Gene Expression Omnibus (NCBI GEO) database. Test (P) and control sample represent sample of JPI-289 treated and control postischemic mouse kidney, respectively. Boxed clusters (clusters 1 and 3) indicate increased clusters in postischemic kidney compared to normal kidney. (**C**) Reactome pathways normalized enrichment score (NES) from gene set enrichment analysis (GSEA) of cluster 1. A positive NES indicates that genes in the JPI-289 treated sample are more expressed than those in the control sample and a negative indicates the opposite. (**D**) Reactome pathways NES from GSEA of cluster 3.

We performed scRNA-seq analysis on proximal tubules (PTs) and endothelial cells (ECs) of postischemic kidney since those cells are difficult to analyze using conventional flow cytometry despite their importance in the pathogenesis of IRI. The increased clusters of PTs in postischemic kidney tissue were identified compared to the normal kidneys of public data (clusters 5 and 8 in Fig. 6A and B). Comparative analysis of postischemic kidney was performed on clusters 5 and 8 of PTs according to the treatment with JPI-289. Compared to the postischemic PTs of the control kidney, the postischemic PTs of the JPI-289 treated kidney expressed higher levels of genes involved in signal transduction, RNA metabolism, translation, and the immune system. The postischemic PTs of the control kidney exhibited greater expression of metabolic, solute carrier-mediated transmembrane transport, and small molecule transport genes compared to the postischemic PTs of the JPI-289 treated kidney (Fig. 6C and D). Moreover, the PTs of the JPI-289 treated postischemic kidney showed increased expression of genes associated with collagen formation and toll-like receptors compared to the PTs of the control kidney (Fig. 6D). The increased clusters of ECs from postischemic kidney tissue were identified compared to the normal kidneys of public data (clusters 1 and 3 in Fig. 7A and B). Compared to the postischemic ECs of the control kidney, the postischemic ECs of the JPI-289 kidney expressed higher levels of genes involved in the transcriptional and translational processes, including RNA polymerase transcription, ribosomal RNA processing, and RNA metabolism, as well as transcriptional regulation by TP3 (Fig. 7C and D). On the other hand, the expression of genes involved in metabolism was enhanced in the postischemic ECs of the control kidney compared to the ECs of the JPI-289 kidney, similar to the results of PTs (Fig. 7D).

### Effects of JPI-289 treatment on the proliferation of hypoxic HK-2 cells

**Figure 8:**
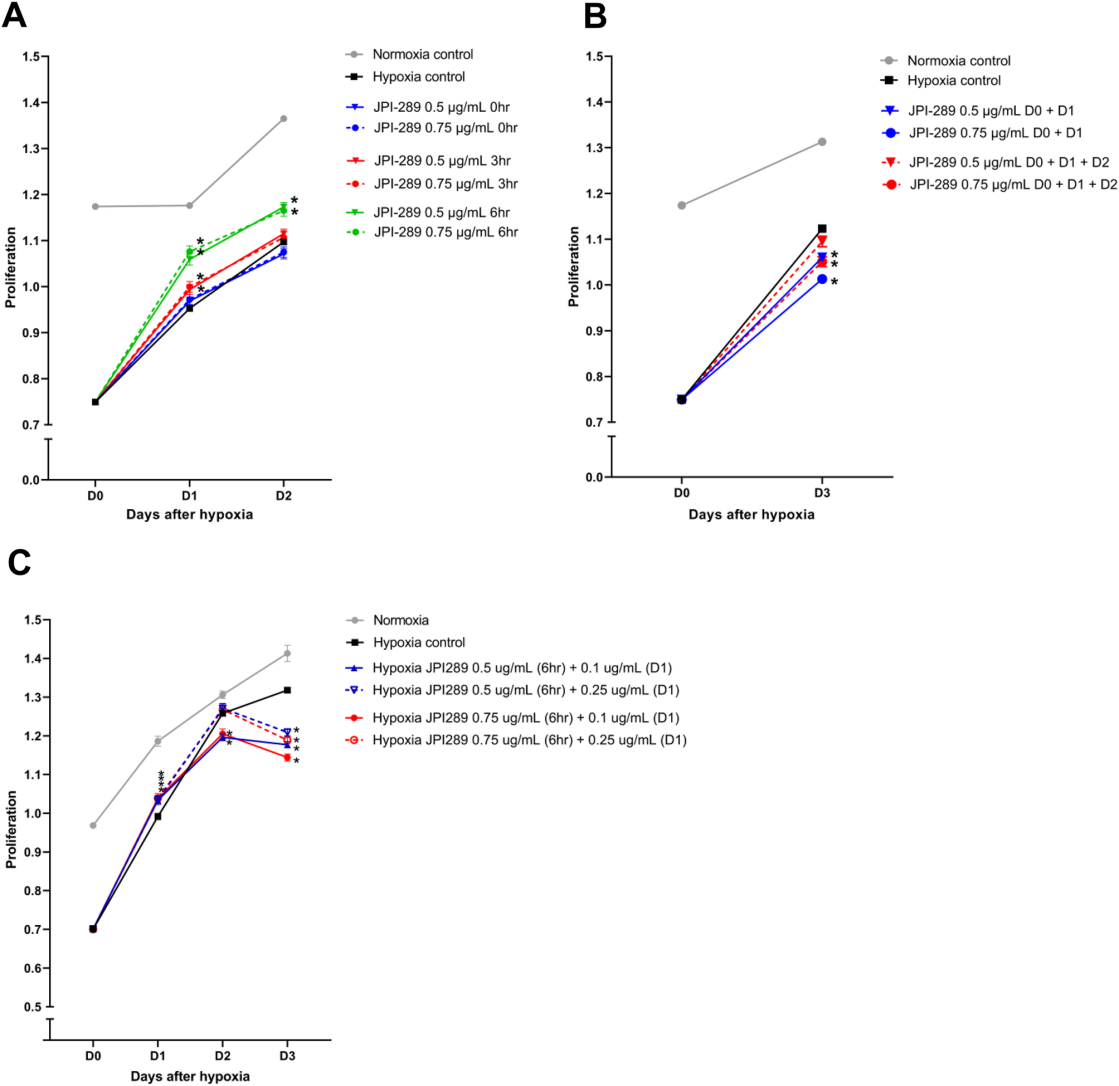
Proliferation of hypoxic HK-2 cells according to the dose and dosing time of JPI-289. (**A**) Treatment with JPI-289 5 or 0.75 µg/mL within 6 h after hypoxia. (**B**) Treatment with JPI-289 5 or 0.75 µg/mL at 6 h and additional same dose at 24 h or 24 and 48 h after hypoxia. (**C**) Treatment with JPI-289 5 or 0.75 µg/mL at 6 h and additional reduced doses 0.1 or 0.25 µg/mL at 24 h after hypoxia.

HK-2 cells were exposed to hypoxia for 48 h and then treated with JPI-289. Figure 8A shows the degree of HK-2 cell proliferation when JPI-289 was administered within 6 h after hypoxia. JPI-289 treatment at 3 h after hypoxia facilitated the proliferation of hypoxic HK-2 cells at 24 h, and JPI-289 treatment at 6 h after hypoxia facilitated proliferation at 24 and 48 h. The time point of the most effective treatment was 6 h, regardless of dose. Figure 8B shows the degree of HK-2 cell proliferation when JPI-289 was administered at 6 h after hypoxia and then additionally administered at 24 h or 24 and 48 h. Additional treatment of JPI-289 after 6 h following hypoxia suppressed the proliferation of hypoxic HK-2 cells. Figure 8C shows when JPI-289 was administered at 6 h after hypoxia and then additional lower doses of JPI-289 were administered at 24 h. JPI-289 treatment at 6 h facilitated the proliferation of hypoxic HK-2 cells at 24 h, but further treatment with JPI-289 suppressed the proliferation even at lower doses.

## Discussion

We previously demonstrated that treatment with a novel PARP inhibitor, JPI-289, immediately after renal IRI induced favorable intrarenal immunologic micromilieu and reduced susceptibility to ischemic AKI. However, the present study revealed that treatment with JPI-289 at 24 h of renal IRI had little effect on the recovery of ischemic AKI until 6 weeks. Furthermore, late treatment with JPI-289 at 24 and 48 h twice after renal IRI induced proinflammatory changes of intrarenal immunologic micromilieu and aggravated tubular injury beyond 6 weeks. The results of the scRNA-seq analysis suggested the potential adverse effects of JPI-289 to aggravate inflammation and promote fibrosis by enhancing gene expression associated with the immune system, toll-like receptors, and collagen formation. HK-2 cells hypoxia model showed that early treatment of JPI-289 facilitated the proliferation of hypoxic HK-2 cells, but late treatment suppressed proliferation. These results suggest that the late treatment with JPI-289 in the healing phase after ischemic AKI has been established may not be effective in promoting renal recovery and may even adversely affect the recovery process.

Poly (ADP-ribose) (PAR), which is a polymer comprising ADP-ribose units, is synthesized from nicotinamide adenine dinucleotide (NAD +) through the enzymatic action of PAR polymerases (PARPs)^16^. These polymerases add poly (ADP-ribose) to proteins as unique posttranslational modifications, and this addition regulates several physiological processes, such as the maintenance of DNA integrity, gene expression, and cell division. PARP-1 is the founding, and best-studied, member of the PARP family, and its activation is important in the DNA damage response and repair of single-stranded breaks^17^. However, overactivation of PARP owing to excessive damage signals causes the regulated necrosis pathway called parthanatos and also activates the classical necroptotic pathway and inflammatory cascades, resulting in cellular death and tissue damage^3,6,18^. It has already been reported in previous studies that pharmacologically or genetically inhibiting PARP can alleviate renal IRI^11,12,19^. Therefore, considering there is no effective treatment for ischemic AKI other than conservative measures, PARP inhibition can be a promising therapeutic candidate.

Our previous study reported that inhibition of PARP-1 by JPI-289 immediately after renal IRI attenuated renal injury and facilitated the proliferation of hypoxic HK-2 cells^15^. The effects of JPI-289 were dose-dependent and improved the intrarenal immunologic micromilieu after renal IRI, leading to attenuation of tubular injury. However, in the present study, the late treatment of JPI-289 at 24 h or later had no effects on renal tubular injury, intrarenal leukocytes trafficking and subtypes, or intrarenal cytokines/chemokines levels until 6 weeks after IRI. Rather, when JPI-289 was additionally administered at 48 h after unilateral IRI, the proportion of atrophic tubules in the postischemic kidney increased at 12 weeks. These results were associated with an increase in total T cells, CD4 T cells, neutrophils, MCP-1, and TNF-α, and a decrease in IL-4, showing proinflammatory changes in intrarenal immunologic micromilieu. Therefore, PARP inhibition at 24 h or later following renal IRI may not improve intrarenal immunologic micromilieu and can impede long-term recovery.

Our findings suggest that the inhibition of PARP may not effectively alleviate renal injury from IRI after PAPR overactivation has been established. PAR generated by PARP is rapidly degraded in cells, with a half-life of a few minutes^20^. Therefore, the effect of PAR depends on the expression of PARP. In the previous study that serially measured the expression of PARP after renal IRI, PARP protein was not detected until 6 h after IRI but was detected from 12 h and continued to be detected until 5 days^19^. Most studies showing the beneficial effect of PARP inhibition have either treated with PARP inhibitor before or within 12 h after tissue injury or used an animal model in which PARP is genetically inactivated^11–14,19,21,22^. Therefore, it can be hypothesized that PARP inhibitors should be administered within 6–12 h after tissue injury to block the adverse effects of PARP overactivation. In a recent study, JPI-289 was administered at 2, 8, 12, and 16 h after induction of cerebral ischemia, and its effectiveness decreased with increasing time to drug administration after ischemia, supporting this hypothesis^14^.

Excessive inhibition of PARP can impair DNA repair and promote cellular damage. Previous studies reported that PARP inhibitors slowed the repair of DNA damage and decreased the survival of cells^23,24^. PARP inhibitors have been studied as a therapeutic target in cancer using these characteristics^25^. DNA repair by PARP does not seem to play a major role in the early injury phase. The animal model in which PARP was genetically inactivated alleviated tissue damage up to 5 days after renal IRI^12^. However, most studies have only evaluated the short-term effects of PARP inhibition, but not the long-term effects^11,12,19^. Furthermore, preemptive or very early treatment in tissue damage is less clinically relevant as a therapeutic agent. Our study evaluated the healing from renal IRI for a long-term follow-up period of up to 12 weeks. The effects of PARP inhibition were not significant until 6 weeks, but at 12 weeks after renal IRI, the renal tubular injury and intrarenal immunologic micromilieu deteriorated. In addition, JPI-289 treatment within 6 h facilitated the proliferation of hypoxic HK-2 cells, but additional treatment after 24 h suppressed proliferation. Therefore, concerns regarding the adverse effects of PARP inhibition on the healing phase of ischemic AKI seem reasonable. It is unclear whether these potential adverse effects of PARP inhibition are because of impaired DNA repair or another effect of PARP inhibition. Further studies are required to elucidate these mechanisms.

According to scRNA-seq analysis, gene expression associated with metabolisms was mainly enhanced in the early stage after IRI. However, PAPP inhibitor enhanced gene expression associated with signaling, transcription, and translation mechanisms of various systems, including the immune system, after IRI. Because excessive PARP activation depletes NAD + and ATP^12^, metabolic activation after IRI may be necessary to compensate for ATP depletion. PARP inhibitors treatment can prevent the depletion of NAD + and keep various cellular functions working. This supports previous reports that treatment with PARP inhibitors in IRI preserved ATP and mitigated renal damage^12,15^. In addition, proteins such as sirtuins, whose functions depend on NAD +, are activated competitively with PARP when NAD + is deficient^26^. Activation of sirtuins was known to alleviate IRI in animal models^27,28^. However, in our study, PARP inhibitors increased gene expressions associated with the immune system, collagen formation, and toll-like receptors after IRI, suggesting that the early benefits of PARP inhibitors treatment may be offset or have detrimental effects during the healing phase.

This study has some limitations. First, the immune system and role of PARP of mice may be different from those of humans, limiting the clinical application. However, similar results were obtained using HK-2 cells derived from human cells in the experiments. Second, the most adequate timing of PARP inhibitor administration after renal IRI is still unknown. Considering the results of previous studies, it may be beneficial to treat PARP inhibitors within 6–12 h after renal IRI. Further studies are required to clarify the most effective timing for the administration of PARP inhibitors in humans. Third, the mechanism of the adverse effects of PARP inhibitors on the late healing phase have not been fully elucidated. In addition to DNA repair, PARP plays various roles such as immune regulation, transcriptional regulation, and post-transcriptional protein modification; therefore, further investigation on the roles of PARP during the healing phase after renal IRI is warranted.

In conclusion, our study demonstrated that the late treatment with PARP inhibitor adversely affected intrarenal immunologic micromilieu and renal tubular injury in the healing phase, impairing the recovery process. Further studies on the most optimal timing and dose of PARP inhibitors are needed to clarify the role of PARP inhibitors as a treatment for ischemic AKI.

## Material and methods

### Animals

Male C57BL/6 mice (9-week-old) were purchased from Orient Bio Inc. (Seongnam, Kyoungki-do, Korea). All the mice were bred in a specific pathogen-free barrier facility. The experiments were approved by the Samsung Medical Center Animal Care and Use Committee (IACUC No. 20200131001 and 20,210,119,003). All methods were performed in accordance with the relevant guidelines and regulations and reported in accordance with ARRIVE guidelines (https://arriveguidelines.org)^29^.

### Murine renal IRI experiments

We evaluated the effects of JPI-289 during the healing phase of two kinds of murine renal IRI models: unilateral or bilateral. Mice were randomized into two groups: control (n = 10) and JPI-289 100 mg/kg (n = 10). In the unilateral IRI model, JPI-289 or saline was injected into the peritoneal cavity once at 24 h or twice at 24 and 48 h after unilateral IRI surgery. Mice were sacrificed at 6 or 12 weeks after surgery. In the bilateral IRI model, JPI-289 or saline was injected into the peritoneal cavity once at 24 h after bilateral IRI surgery. Mice were sacrificed 6 weeks after surgery. JPI-289, 10-ethoxy-8-(morpholinomethyl)-1,2,3,4-tetrahydrobenzo [h] [1, 6] naphtyridin-5(6H)-one dihydrochloride dehydrate was synthesized, and supplied by Jeil Pharmaceutical Co. Ltd. (Seoul, Korea).

Mice were anesthetized by injecting ketamine (100 mg/kg; Yuhan, Seoul, Korea) and xylazine (10 mg/kg; Bayer, Leverkusen, Germany) into the peritoneal cavity. For bilateral renal IRI, the renal pedicles of both kidneys were carefully isolated and clamped for 27 min with a microvascular clamp (Roboz Surgical Instrument, Gaithersburg, MD) through an abdominal midline incision. For unilateral renal IRI, the renal pedicle of the left kidney was isolated and clamped for 45 min using the same technique. Mice were well-hydrated with warm sterile saline of 40 °C on a temperature-controlled heating table of 45.5 °C. In all animal experiments, the same methods for maintaining body temperature were applied to both groups. Mice recovered after surgery with no restriction on food and water. Mice were sacrificed at 6 and 12 weeks after surgery and kidneys were harvested after exsanguination. All mice survived until the time of prescheduled sacrifice after unilateral IRI, but one of the 20 mice died prematurely after bilateral IRI.

### Assessment of renal function

Plasma creatinine (Arbor Assays, Ann Arbor, MI) and blood urea nitrogen (BUN; Fujifilm, Bedford, UK) concentrations were measured using colorimetric kits according to the manufacturer’s instructions. In the unilateral IRI model, cystatin C (R&D Systems, Minneapolis, MN) was additionally measured. Baseline BUN, plasma creatinine, and cystatin C levels were measured 7 days prior to surgery. Mice plasma samples were taken serially via tail veins until sacrifice.

### Tissue histological analysis

Postischemic kidney tissue sections were fixed with 10% buffered formalin. Hematoxylin and eosin (H&E) or Masson's trichrome staining were used to evaluate the histopathological changes of postischemic kidneys. A renal pathologist blinded to the experimental groups evaluated the extent of renal tubular damage, necrosis, or atrophy on H&E-stained slides and the extent of interstitial fibrosis on Masson's trichrome-stained slides.

### CD45 immunohistochemistry and tissue FAXS analysis

Immunohistochemical staining of a cluster of differentiation (CD) 45 (leukocyte common antigen) using formalin-fixed renal tissue sections was performed with reference to a previous report^30^. The renal tissue sections (4-µm-thick) were deparaffinized using xylene, rehydrated in a graded alcohol series, and then transferred to citrate buffer solution (pH 6.0). The slides were placed in a pressure cooker and heated in microwave for 10 min to enhance antigen retrieval. After cooling, the slides were immersed in hydrogen peroxide solution (Dako, Carpinteria, CA) for 30 min to block the endogenous peroxidase activity. After overnight incubation at 4 °C with serum-free protein block (Dako), the slides were incubated at room temperature for 1 h with a 1:100 dilution of monoclonal rat anti-mouse antibody to CD45 (BD Biosciences, San Jose, CA). After being rinsed, the CD45-stained sections were incubated for 30 min with a secondary antibody using a Dako REAL EnVison kit (Dako) at room temperature. Staining with 3,3′-diaminobenzidine tetrahydrochloride (Dako) was performed on the slides to extract brown color, then counterstaining with Mayer's hematoxylin (Dako) was performed. A TissueFAXS workstation (Tissue Gnostics, Vienna, Austria) was used to analyze and calculate the percentages of CD45-positive cells infiltrated into renal tissues, as described previously^30^.

### Flow cytometric analysis of kidney-infiltrating mononuclear cells

KMNCs from kidney tissues were isolated based on an established protocol^31^. Briefly, decapsulated kidneys were immersed in Roswell Park Memorial Institute buffer (Mediatech, Manassas, VA) containing 5% fetal bovine serum and disrupted mechanically using a Stomacher 80 Biomaster (Seward, Worthing, UK). Samples were strained, washed, and resuspended in 36% Percoll (Amersham Pharmacia Biotech, Piscataway, NJ), followed by gentle overlaying onto 72% Percoll. After centrifugation at 1,000 × *g* for 30 min at room temperature, KMNCs were collected from the interface of 36% and 72% Percoll.

Isolated KMNCs were resuspended in flow cytometry staining buffer and preincubated with anti-CD16/CD32 antibodies for 10 min to minimize nonspecific binding through Fc-receptors. KMNCs were incubated with anti-mouse anti-CD3, CD4, CD8, CD19, CD21, CD25, CD44, CD45, CD62L, CD69, CD126, CD138, Gr-1, F4/80, FoxP3, and NK1.1 (all were acquired from BD Biosciences, San Jose, CA) for 25 min at 4 °C, washed with FACS buffer, and fixed with 1% paraformaldehyde solution. Samples were acquired using a BD FACSVerse flow cytometer. Data were analyzed using the FlowJo v10 software (BD Biosciences).

### Multiplex cytokine/chemokine assay

Multiplex cytokine and chemokine analysis in whole kidney protein extracts was conducted using an R&D Mouse Magnetic bead-based multiplex assay for the Luminex® platform (LXSAMSM-08) (R&D Systems, Minneapolis, MN). This multiplexed, particle-based, flow cytometric assay uses anti-cytokine monoclonal antibodies linked to microspheres incorporating the distinct properties of two fluorescent dyes. We measured monocyte chemoattractant protein (MCP-1/CCL2), regulated on activation, normal T cell expressed and secreted (RANTES/CCL5), interferon-gamma (IFN-γ), interleukin (IL)-4, IL-6, IL-10, tumor necrosis factor-alpha (TNF-α), and vascular endothelial growth factor (VEGF). The limit of detection values for each cytokine/chemokine were as follows: IL-2, 1.0 pg/mL; IL-4, 0.4 pg/mL; IL-6, 1.1 pg/mL; IL-10, 0.8 pg/mL; IFN-γ, 1.1 pg/mL; CCL-2, 6.7 pg/mL; CCL-5, 2.7 pg/mL; TNF-α, 2.3 pg/mL; VEGF 0.3 pg/mL. The value of each cytokine or chemokine was normalized by dividing the raw protein concentration (mg/mL, measured using a Pierce BCA protein assay kit, Thermo Fisher Scientific, Waltham, MA), and the final unit of cytokine/chemokine value was expressed as “pg/mg”.

### Single cell RNA-sequencing and data analysis

We performed a separate experiment for single-cell RNA sequencing (scRNA-seq) analysis of post-ischemic kidneys. JPI-289 100 mg/kg (n = 1) or saline (n = 1) was injected into the peritoneal cavity immediately before reperfusion during bilateral IRI surgery. After sacrifice at 24 h following bilateral IRI surgery, samples were immediately transferred for tissue preparation.

Each cell suspension was processed for the generation of scRNA-seq libraries using the Chromium Next Gem Single cell 5′ kit v2 (10 × Genomics, USA, 1,000,263) according to the manufacturer’s protocol, and sequencing libraries were generated using Illumina HiSeq2500 according to the manufacturer’s instructions (Illumina). Both reads were aligned to the mm10-2020-A mouse genome reference sequence and quantified using CellRanger (version 5.0.1-dirty).

Publicly available datasets of adult mouse normal kidney samples of GSE107585 and GSE157079 were downloaded from the National Center for Biotechnology Information Gene Expression Omnibus (NCBI GEO) database and integrated with our sequencing data using Seurat v4.1.0 (Seurat v4 is released under the open source GPLv3 license, and all code is available at https://www.github.com/satijalab/seurat.) As a result, a total of 72,010 cells were used for analysis (Supplementary Fig. S5). Cells with more than 50% mitochondrial gene expression in unique molecular identifier (UMI) counts were excluded from the analysis. For batch correction, the harmony R package was used. For variable genes, 2000 genes were selected among genes with a mean expression of 0.1 or more and 8 or less for each sample. The major cell types were annotated by comparing the canonical marker genes^32,33^. To identify differentially expressed genes (DEG) between two groups, we used the Seurat FindMarkers function with the Wilcoxon rank sum test.

### HK-2 cell hypoxia model and proliferation assay

Human kidney (HK)-2 cells (an immortalized proximal tubule epithelial cell line from a normal adult human kidney) were used for an in vitro tubular cell hypoxia model to investigate the effects of JPI-289 on ischemic AKI. HK-2 cells were purchased from the American Type Culture Collection (CRL-2190, Manassas, VA) and cultured in keratinocyte serum-free media (Thermo Fisher Scientific) supplemented with bovine pituitary extract and human recombinant epidermal growth factor. Cells were incubated at 37 °C in a humidified atmosphere of 5% CO_2_, with a change of medium every 2–3 days. HK-2 cells were exposed to 1% O_2_ and 5% CO_2_ balanced with nitrogen in a multi-gas incubator (APM-30D, Astec, Fukuoka, Japan) for 48 h to induce hypoxia and then were taken out. According to the manufacturer’s instructions, the degree of HK-2 cell proliferation was measured using a Cell Titer96 aqueous one solution cell proliferation assay (Promega, Madison, WI).

The two control groups were the normoxia (21% O_2_) and hypoxia (1% O_2_) control groups. We performed three sets of experiments according to the dose and time of JPI-289 treatment after hypoxia. In the first experiment, hypoxic HK-2 cells were treated with 0.5 or 0.75 µg/mL of JPI-289 at 0, 3, or 6 h after hypoxia, and proliferation was measured at 24 and 48 h after hypoxia. In the second experiment, hypoxic HK-2 cells were treated with 0.5 or 0.75 µg/mL of JPI-289 at 6 h after hypoxia and further treated with the same dose of JPI-289 at 24 h or 24 and 48 h, and proliferation was measured at 72 h after hypoxia. In the third experiment, hypoxic HK-2 cells were treated with 0.5 or 0.75 µg/mL of JPI-289 at 6 h after hypoxia and further treated with 0.1 or 0.25 µg/mL of JPI-289 at 24 h, and proliferation was measured at 24, 48, and 72 h after hypoxia.

### Statistical analysis

All data were expressed as mean ± standard error of the mean or median (interquartile range). Differences between groups or time points were analyzed using the Mann–Whitney U-test. All statistical analyses were conducted using GraphPad Prism version 8 (GraphPad Software, La Jolla, CA). A two-sided *P* value < 0.05 was considered statistically significant.

### Supplementary Information

Supplementary Figures.

## Data Availability

The scRNA-seq datasets generated and/or analyzed during the current study are available in the NCBI GEO repository (GSE239905). The dataset used during the current study is available from the corresponding author on reasonable request.
