# Supplementary material for: Effects of poly (ADP-ribose) polymerase inhibitor treatment on the repair process of ischemic acute kidney injury
Source: Sci Rep. 2024 Jan 2;14:159. doi: 10.1038/s41598-023-50630-2 (PMC10761972; doi:10.1038/s41598-023-50630-2)
Supplement: Supplementary file 1 — Supplementary Figures. [file 41598_2023_50630_MOESM1_ESM.pdf]

Supplementary Information

Effects of Poly (ADP-ribose) Polymerase Inhibitor Treatment on the Repair Process of Ischemic Acute Kidney Injury

Junseok Jeon[1], Kyungho Lee[1], Hye Ryoun Jang[1], Kyeong Eun Yang[2], Cheol-Jung Lee[2], Hyeonju Ahn[3], Woong-Yang Park[3,4], Jung Eun Lee[1], Ghee Young Kwon[5], Yoon-Goo Kim[1], Wooseong Huh[1]*

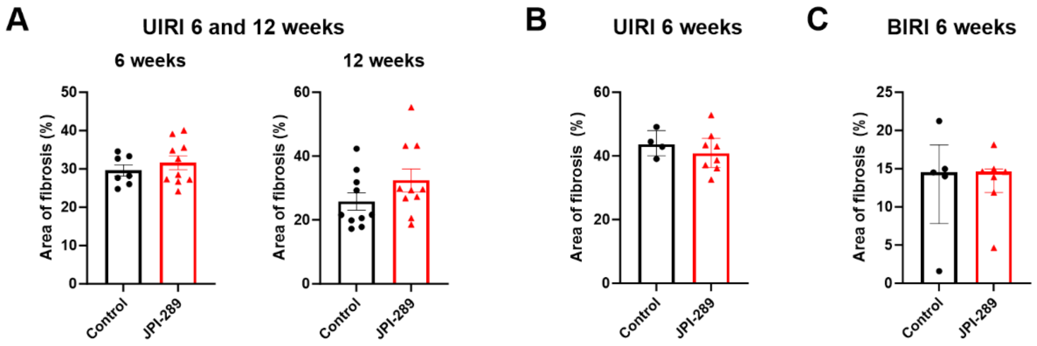

Supplementary Fig. S1. Extent of renal tissue fibrosis after renal IRI assessed using Masson's trichrome stain from postischemic kidney. (A) Treatment with JPI-289 100 mg/kg at 24 and 48 h twice versus control after unilateral IRI. (B) Treatment with JPI-289 100 mg/kg at 24 h once versus control after unilateral IRI. (C) Treatment with JPI-289 100 mg/kg at 24 h once versus control after bilateral IRI.

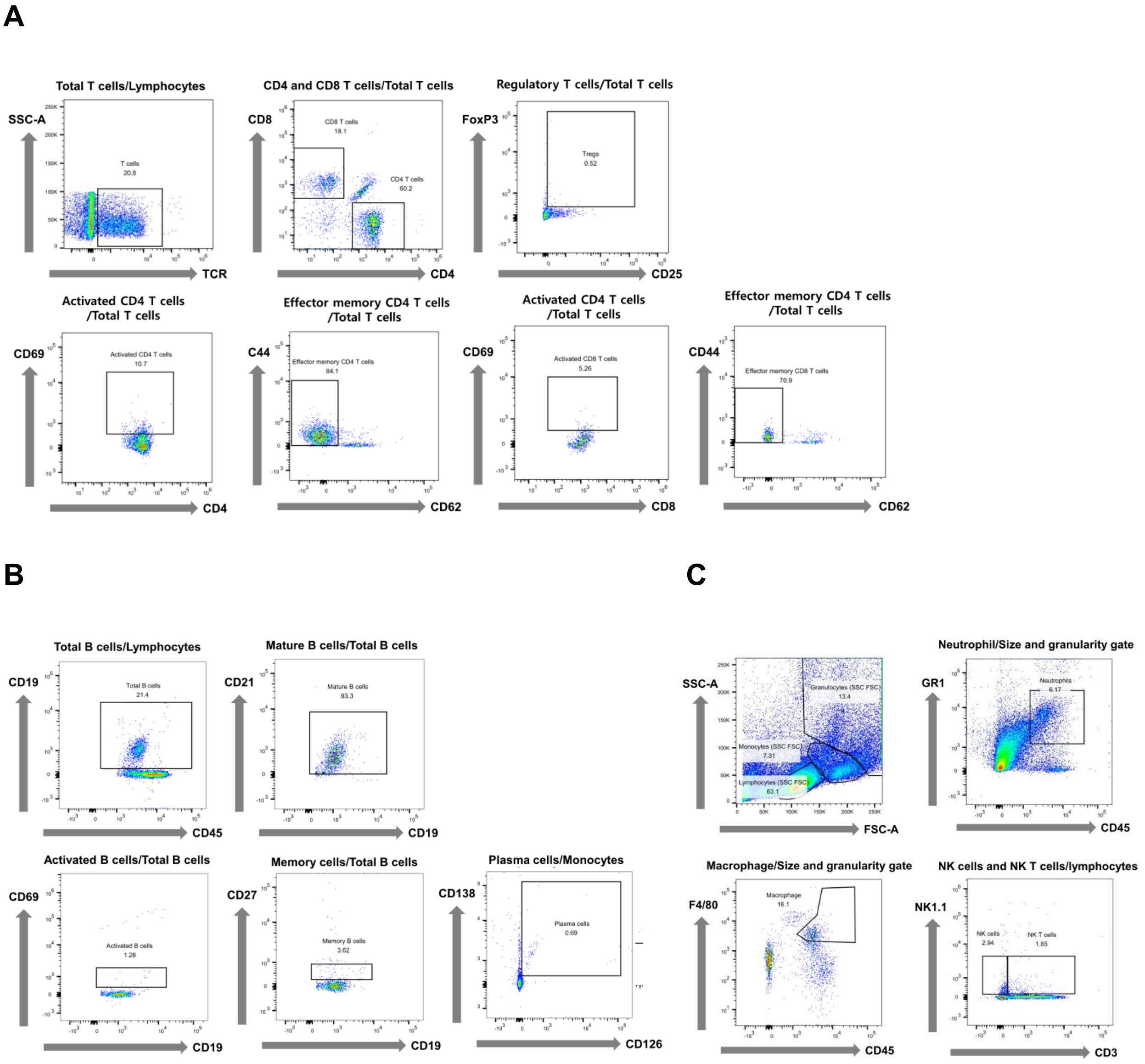

Supplementary Fig. S2. Flow cytometry analyses of KMNCs isolated from postischemic kidneys. (A) T cell subtypes, (B) B cell subtypes, and (C) Neutrophil, macrophage, NK cells, and NK T cells.

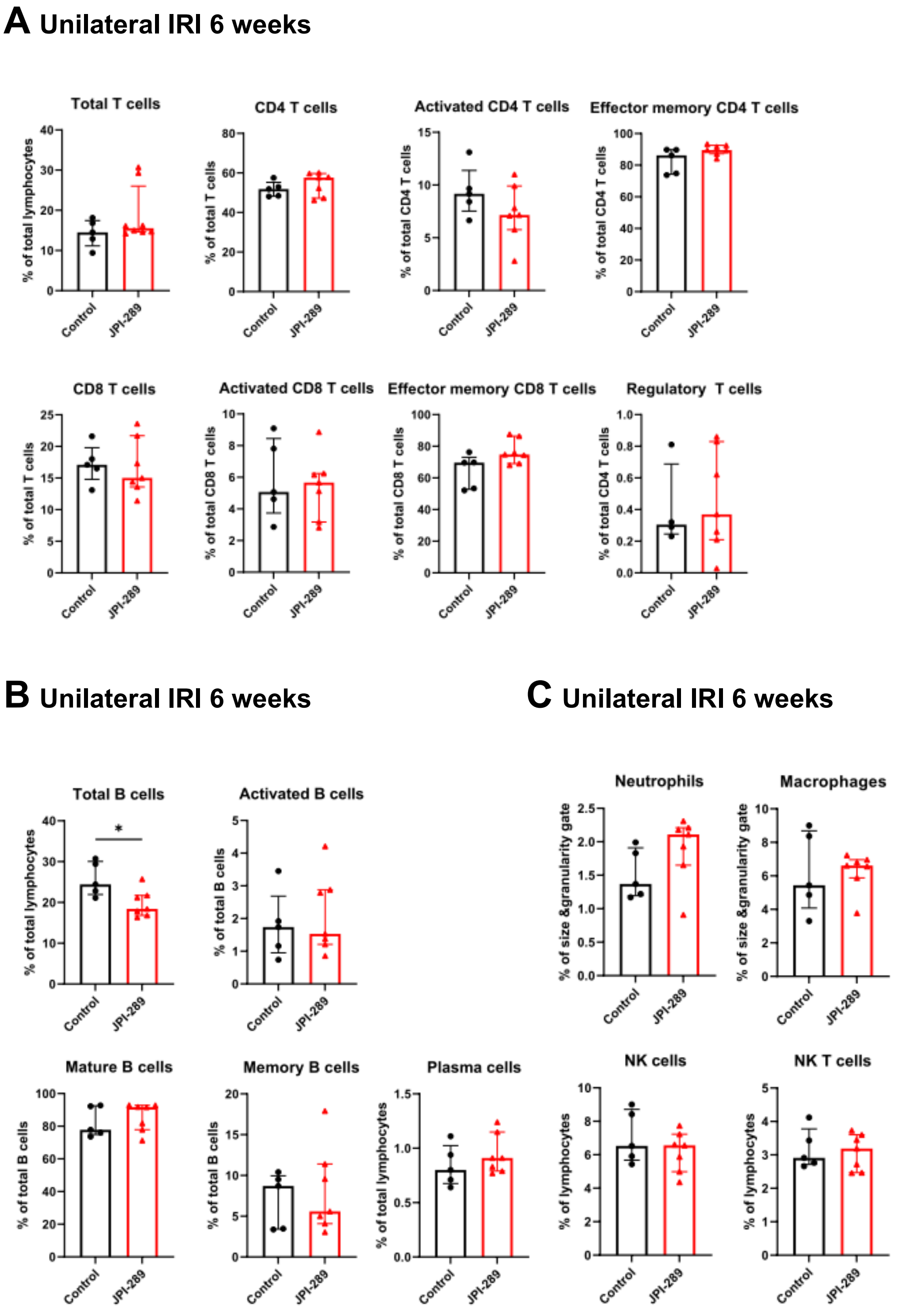

Supplementary Fig. S3. Flow cytometry analyses of intrarenal leukocyte subtypes between treatment with JPI-289 100 mg/kg at 24 h versus control after unilateral IRI. (A) T cell subtypes, (B) B cell subtypes, (C) Neutrophils, Macrophages, NK cells, and NK T cells

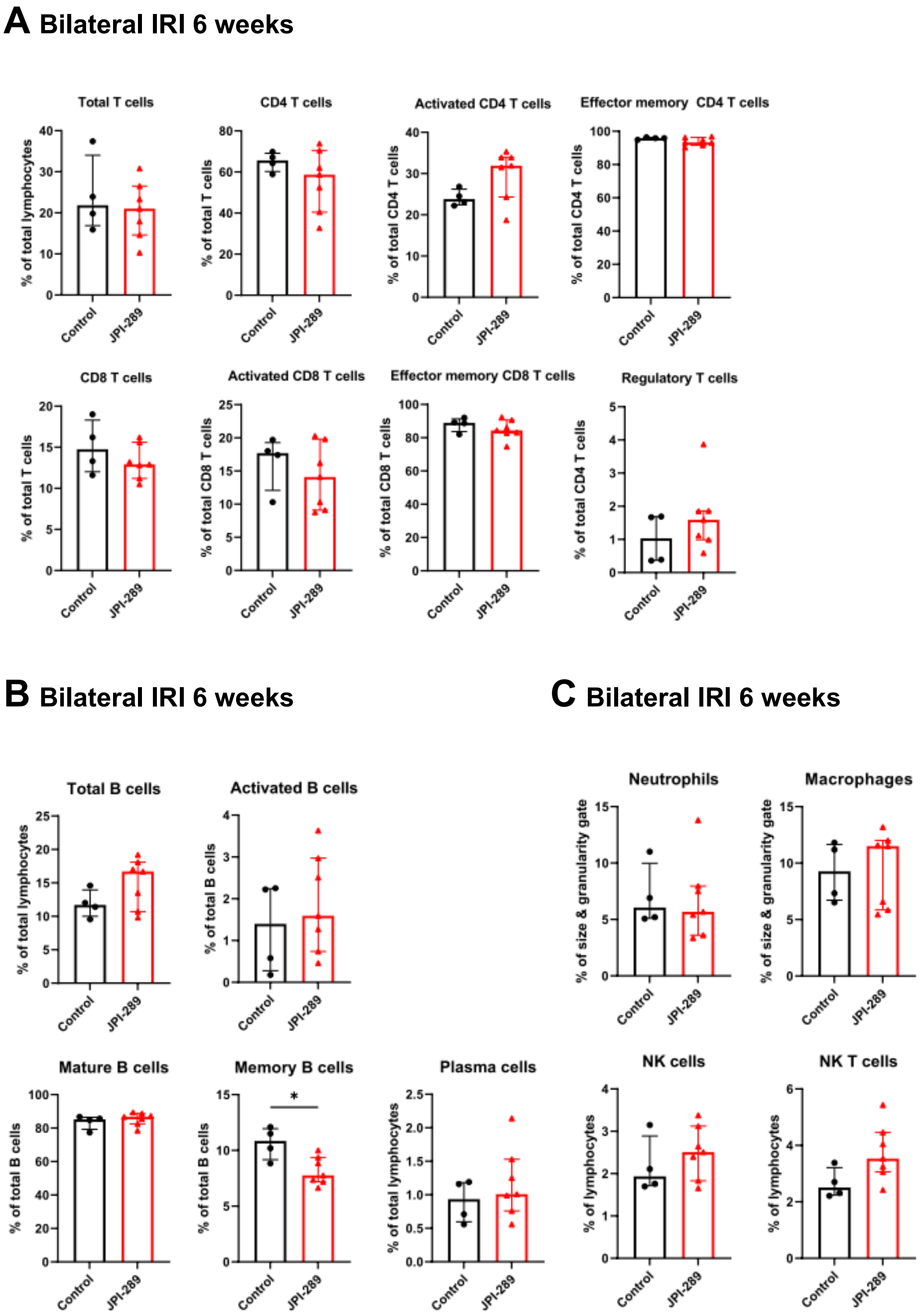

Supplementary Fig. S4. Flow cytometry analyses of intrarenal leukocyte subtypes between treatment with JPI-289 100 mg/kg at 24 h versus control after bilateral IRI. (A) T cell subtypes, (B) B cell subtypes, (C) Neutrophils, Macrophages, NK cells, and NK T cells

A

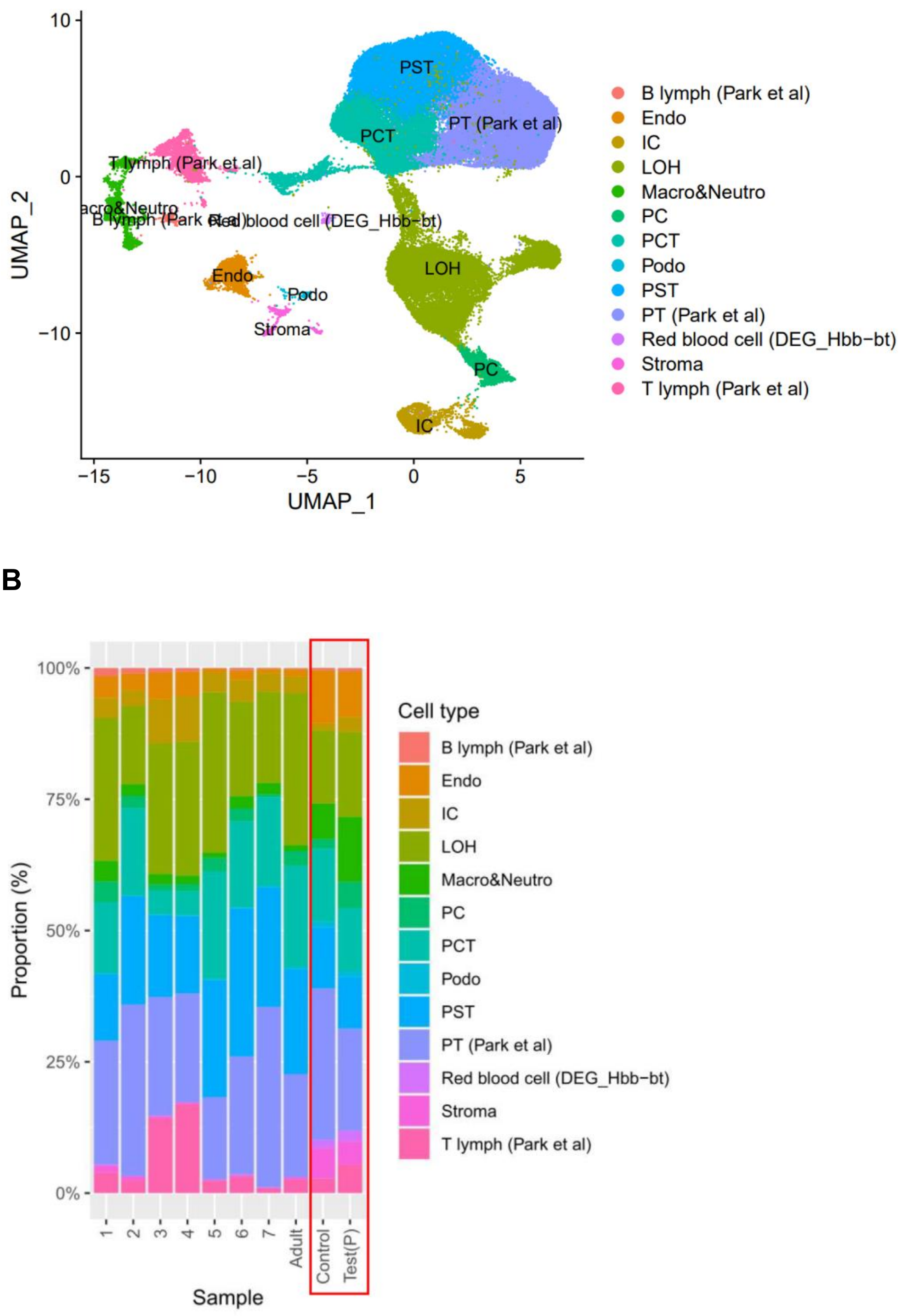

Supplementary Fig. S5. Diversity and proportion of cell types in the scRNA-seq results for mouse kidney tissues (A) UMAP embedding of scRNA-seq profiles of mouse kidney tissues, colored according to cells. (B) Proportions of cells of different subsets for each sample. Samples 1–7 and adult samples are mouse normal kidney samples of GSE107585 and GSE157079 acquired from the National Center for Biotechnology Information Gene Expression Omnibus (NCBI GEO) database. Test (P) and control sample represent sample of JPI-289 treated and control post-ischemic mouse kidney, respectively.
